# Effect of Alginate Coating and Modified Atmosphere Packaging on the Stability of Fresh-Cut Guava Shells (*Psidium guajava* L.)

**DOI:** 10.1021/acsomega.5c08483

**Published:** 2025-11-13

**Authors:** Marcela Rangel-Marrón, Ricardo H. Hernández-Figueroa, Aurelio López-Malo, Emma Mani-López

**Affiliations:** † Departamento de Ingeniería Química y Alimentos, 27806 Universidad de las Américas Puebla, San Andrés Cholula, Puebla 72810, Mexico; ‡ Facultad de Química, 27780 Universidad Autónoma del Carmen, Ciudad del Carmen, Campeche 24158, Mexico

## Abstract

This study aimed to evaluate the effect of alginate-based edible coating (EC) and modified atmosphere (MA) packaging on fresh-cut guava shells to preserve them during refrigerated storage. Alginate films (AF) were formulated and characterized. The gas composition, titratable acidity (TA), total soluble solids (TSS), ascorbic acid (AA), color, firmness, pectin-esterase enzymatic activity, and microbiological quality were determined for 15 days at 5 °C. AF’s physicochemical, mechanical, and barrier properties were measured. The fruit was sanitized and cut to remove seeds. Then, it was packaged under the following conditions: (a) atmospheric air without EC (control), (b) fruit in MA packaging, and (c) coated guava shells with MA packaging (EC + MA). AF exhibited adequate properties to form EC. EC + MA packaging slowed fruit maturation and preserved the ascorbic acid content better at the end of storage than MA packaging and control fruits. After 10 days of storage, the fruits luminosity was similar to the initial values. EC + MA maintained the fruit’s firmness for up to 15 days. The pectin-esterase activity was not affected by MA packaging or EC + MA during storage. After 15 days of storage, TMAB were under 10^6^ CFU/g in fresh-cut guava shells packaged in MA or EC + MA. Considering the physicochemical quality and microbial counts, fresh-cut guava shells with EC + MA were preserved for 10 days at 5 °C; thus, this treatment potentially could reduce fruit waste.

## Graphical abstract

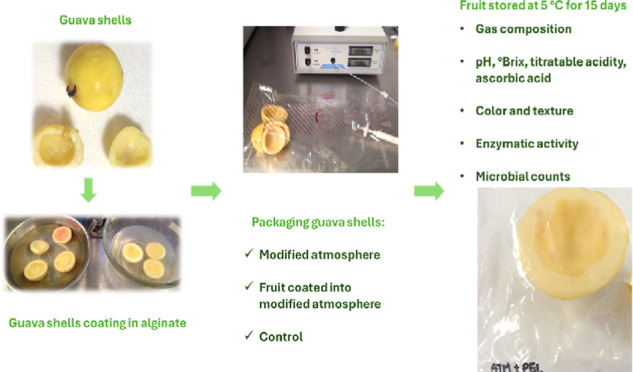

## 1 Introduction

Guava (*Psidium guajava* L.) is a subtropical fruit native to southern Mexico and Central America; it is currently produced in tropical and subtropical regions and is of commercial interest worldwide. Guava exhibits a fruit-sweet-sour flavor, typically with a pleasant scent (sweet-fresh); it is a climacteric fruit with intense respiratory activity.^1^ Its bioactive compounds are primary carotenoids, flavonoids, and polyphenols.^2^ Mexico was the fifth guava producer in the world, with 0.7 million tons in 2023,^3^ below countries such as India, Pakistan, Brazil, and Egypt. Currently, there is a growing demand for fresh-cut fruits ready-to-eat, which are attractive products because of their nutritional value and sensory properties (color, odor, and flavor). Fresh-cut fruits are highly perishable and exhibit a few days of shelf life under cold storage. Thus, the application of adequate hurdle technology could extend their shelf life.

During processing, cutting the fruit damages tissue and cellular integrity, leading to increased enzymatic, respiratory, and microbial activity that reduces shelf life.^4^ The modified atmosphere (MA) technology has allowed fresh-cut produce to reach the market.^5^ MA technology uses low oxygen (O_2_) and/or high carbon dioxide (CO_2_) to slow degradation processes during storage. With low temperatures, MA can reduce the respiration rate, provide a product moisture barrier, minimize the action of spoilage microorganisms, and stop the darkening and dehydration of the cut surface, impacting the final product.^6,7^ For minimally processed guavas, MA combined with osmotic dehydration and cold storage showed positive results as fruit quality was maintained for 24 days.^8^

Edible coatings (EC) could be a good alternative or complementary to MA packaging to extend the shelf life of fresh-cut fruits. EC provides a semipermeable barrier to gases and water vapor, which reduces respiration and enzymatic darkening.^9^ Sodium alginate is a natural polysaccharide derived from marine brown algae with a backbone of (1–4) linked β-d-mannuronic acid (MA) and α-l-guluronic acid (GA), which form irregular patterns of GA-GA, MA-GA, and MA-MA blocks.^10^ The GA blocks can interact with divalent calcium ions to form gels through cross-linking. Sodium alginate is hydrophilic, biocompatible, renewable, biodegradable, and has relatively low production cost; these characteristics are advantages^11^ over other polysaccharides in EC formulations, thus, it is widely studied in food applications. These properties position alginate as a sustainable and environmentally friendly material. In addition, alginate’s unique gelling, thickening, and film-forming properties, as well as its interaction with calcium ions, result in semipermeable membranes with excellent potential in food preservation.^12,13^ Alginate cross-linked membranes (coatings) maintain the balance of CO_2_ and O_2_ during respiration (vital quality of fresh produce), and the reduced gas exchange prevents discoloration, retains texture, and preserves or enhances the appeal.^13^ Sodium alginate EC in foods acts as a barrier to water vapor and oxygen, thereby helping to preserve food freshness, enhance visual appeal through transparency, and demonstrate robust mechanical properties. At the same time, fruits keep moisture.^14^ For instance, on fresh-cut guava, halves guava coated (1% cashew gum, 2% CMC, and 1% glycerol) delayed fungal growth, weight loss, firmness loss, and browning for 8 days at 25 °C;^15^ guava cubes maintained its weight, phenolic compounds, ascorbic acid, firmness, color, and sugars when coated using flaxseed protein (5%) + guar gum (1%);^16^ chitosan (1%) coatings on “Paluma” guava rings or alginate enriched with acemannan coatings on guava pieces preserved the physicochemical, sensory, and functional properties for 9 or 12 days at 6 or 3 °C.^2,17^ Potato starch (3%) + chitosan (0.5%) extended the shelf life of guava slices for 18 days, stored at 4 °C.^18^ Chitosan (1%) was the most effective EC (between alginate, pectin, carboxymethylcellulose, carrageenan, and starch) to preserve the quality of fresh-cut guavas for 4–5 days at 5–7 °C more than control fruits.^19^ These studies reveal that EC improves the fresh-cut guava quality without affecting its physicochemical and microbiological quality. Selected authors have used alginate-based EC plus MA packaging in tropical fruits as a strategy to prolong the fresh-cut shelf life, such as kiwifruit,^20^ papaya,^21^ and pineapple.^22^ They showed that alginate-based EC combined with MA packaging decreased respiratory activity, maintained physicochemical properties, and presented lower microbial growth. However, there is limited information on the combined effect of EC and MA packaging on fresh-cut guava shells. Therefore, this research aimed to assess the effect of alginate-based EC and MA packaging on fresh-cut guava shells to preserve them during storage at 5 °C. Also, alginate films (AF) were formulated and characterized by physicochemical, mechanical, and barrier properties. Uncoated guava shells in atmospheric air packaging (control), uncoated into MA packaging (MA), and alginate-EC into MA packaging (EC + MA) were prepared and stored at 5 °C for 15 days. Gas composition, physicochemical properties, pectinesterase activity, and microbial quality were analyzed in the fruits during storage. During storage, guava shells with EC and MA packaging retained better nutritional and quality properties. Further studies on the sensory evaluation of guava shells during storage should be performed, and assessing active-EC (antioxidant, antimicrobial, or antibrowning) is necessary to improve the preservation of guava shells.

## 2 Results and Discussion

### 2.1 Alginate Films Characterization

AF’s physicochemical, mechanical, and barrier characteristics are essential to their effectiveness as edible fruit coatings. The films thickness is directly associated with mechanical, physical, and barrier properties. The thickness of alginate films was 69 ± 2 μm. Films intended for fruits EC are desirable because they are transparent and do not alter the fruits’ color. AF were transparent and had a Lh = 21.74 ± 0.05, *h* = 181.25° ± 0.06, and *C* = 1.30 ± 0.03. Similar thickness and color parameters were reported by Hernández-Figueroa et al.^23^ for AF. Puncture resistance and elongation demonstrate mechanical integrity and flexibility, allowing the coating to maintain adhesion. The puncture resistance (0.351 ± 0.118 N) and elongation (17.55 ± 2.60%) of the AF were aligned with those reported by Hernández-Figueroa et al.^23^ for elongation (22.97%) and lower than the puncture resistance (1.06 N) reported by Comaposada et al.^24^ The water vapor permeability (WVP) determined the coating’s ability to control weight loss. The film’s WVP was 5.73 ± 0.87 g mm/h kPa m^2^. Slightly lower values of WVP (0.37 to 1.38 g mm/h kPa m^2^) were reported for AF formulated with different levels of alginate (6–14%), glycerin (5–25%), carboxymethyl-cellulose (0–0.7%), immersion times in CaCl_2_ solution (4–20 min), and concentrations of CaCl_2_ solution (2–10%) (Zhang et al.);^25^ differences can be associated with films’ biopolymers composition and higher concentrations. Lower WVP values indicate that the films present better moisture barriers. Films moisture content was 11.87 ± 2.64% and water solubility (WS) was 45.99 ± 1.58%. Low moisture content was expected in films due to the drying process. WS is associated with water resistance and stability; thus, WS defines the potential applications of films. Calcium ions concentration is directly involved with WS because determining the film cross-linking, calcium makes the intermolecular bonds more cohesive and arranged.^25^ Calcium solutions of 2–3% are considered optimal for low WVP and WS.^25,26^ Higher moisture content (20%) and WS (65%) were previously recorded by Janik et al.^27^ for AF enriched with chestnut extract. Panahirad et al.^28^ reported that these studied features help in understanding the structure–function relationships of alginate when forming coatings and films.

### 2.2 Surface Solid Density of Coatings

The integrity of coatings is a key factor in fresh-cut fruits because protective properties are directly associated with them and depend on film flexibility, surface tension, and adherence to the food product.^29^ Therefore, the indicator EC’s average thickness on guava shells was estimated using the surface solid density (SSD). The SSD for guava shells was 18.06 × 10^–3^ ± 0.0019 g/cm^2^ (0.18 g/m^2^). EC contributed to the guava shell mass at ∼10.5%. Lower values of SSD were reported for EC formulated with papaya puree-alginate and variable concentrations of citric acid (4.46 to 7.28 × 10^–3^ g/cm^2^).^29^ Higher values were reported by Medina-Jaramillo et al.^30^ for alginate EC at 2% on blueberries (3.7 g/m^2^). Primary differences were attributed to each study’s alginate and total solids concentration.

### 2.3 Gas Composition

The O_2_ and CO_2_ gas composition of the guava-shells packages during the storage is presented in Figure 1a,b. Gas composition changes are expected due to fruit respiration (involves the enzymatic oxidation of organic molecules such as carbohydrates, sugar, starch, fatty acids, and proteins). In addition, bag permeability impacts the internal gas composition. After 5 days, O_2_ concentration declined, and CO_2_ increased in the headspace of bags containing the control fruits due to respiration. Reported respiration rates for guava under low-temperature storage vary depending on cultivar and methodology. Bron et al.^31^ observed rates of 0.16 to 0.43 mmol CO_2_/kg h between 1 and 11 °C. Similarly, Yadav et al.^32^ reported values of 10–20 mg CO_2_/kg h at 5 °C and 40–80 mg CO_2_/kg h at 20 °C, which align with typical ranges for tropical fruits stored in refrigeration. Modeling studies by Mangaraj and Goswami^33^ confirmed respiration rates within comparable magnitudes. Recent reviews of postharvest physiology also emphasize that guava generally exhibits moderate respiration compared with other climacteric tropical fruits, supporting these estimates.^34^ The selected film had an oxygen permeability of approximately 4333 cm^3^/m^2^ day atm at 23 °C, which remains relatively high even at 5 °C. Based on literature reports, guava and related tropical fruits exhibit respiration rates in the range of 10–25 mL O_2_/kg h at 5 °C. Considering these respiration values and the guava shells’ mass, it is possible to estimate the O_2_ consumption between 12 and 30 cm^3^/day. Although the fruit consumes O_2_, the high film permeability allows O_2_ ingress at a comparable magnitude, preventing a stable low-O_2_ environment from developing. As a result, the O_2_ concentration increased (from 5 to 12%) and CO_2_ decreased (from 3.9 to 1.1%) after 5 days, indicating failure to maintain the intended MA. Assuming a headspace-to-fruit ratio of ∼1:20 (v/v) and the given film area, the estimated O_2_ influx through the film would exceed the O_2_ consumption by the fruit.

**Figure 1:**
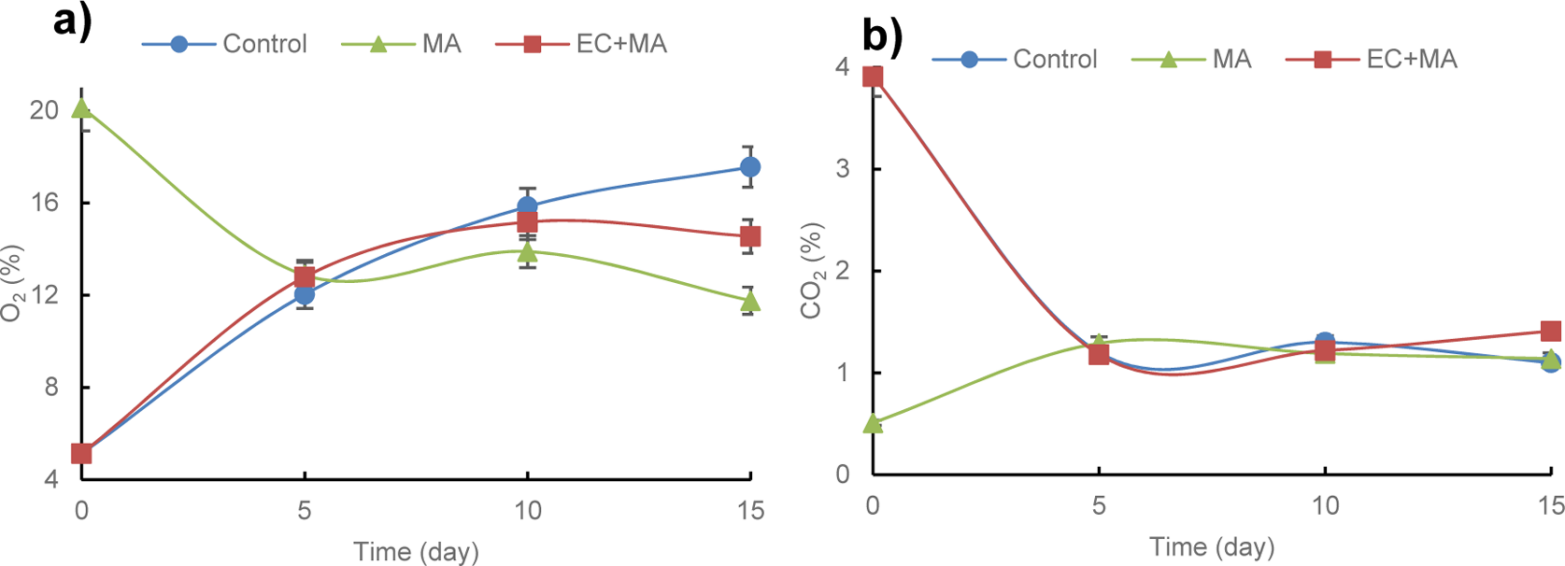
Gas composition, (a) oxygen and (b) carbon dioxide inside the plastic bags containing fresh-cut guava shells in modified atmosphere (MA) packaging and alginate-based coating plus MA packaging (EC + MA) during the refrigerated (5 °C) storage. Control fruit was packaged with air and uncoated.

In contrast, the EC + MA treatment exhibited a more stable gas composition, with O_2_ levels stabilizing at ∼14% and CO_2_ around 1–2% from days 5 to 15. This stability suggests that the edible coating (EC) provided an additional gas diffusion barrier, lowering the respiration rate of the fruit and partially compensating for the high permeability of the packaging film. Alginate-based EC + MA packaging inhibited higher respiration rates of fresh-cut pineapple than control or EC or MA packaging alone, stored at 4 °C.^22^ The reducing respiration rate effect of alginate EC on whole guavas has been reported previously by Nair et al.^35^

### 2.4 Physicochemical Properties of Guava Shells

Table 1 shows the titratable acidity (TA), total soluble solids (TSS, °Brix), and ascorbic acid (AA) content of guava shells packaged with MA, EC + MA, and control during storage at 5 °C. Slight decreases in TA (<0.05%) were observed during the storage and between the guava treatments. Low temperature contributes to maintaining the TA of guava shells by slowing the respiration rate. Other studies have shown higher reductions in TA (∼0.15%) after 12 days of storage (4 °C) of fresh-cut guava cubes coated with flaxseed protein + guar gum + Tween 40 and uncoated fruit.^16^

**Table 1:** Physicochemical Properties of Fresh-Cut Guava Shells in Modified Atmosphere (MA) Packaging and Alginate-Based Coated Plus MA Packaging (EC + MA) Stored at 5 °C^a^ ^,^ ^b^

parameter	time (day)	control	MA	EC + MA
titratable acidity (%)	0	0.67 ± 0.04 aA	0.67 ± 0.03 aA	0.60 ± 0.04 aA
	5	0.57 ± 0.07 aB	0.54 ± 0.04 aB	0.57 ± 0.07 aA
	10	0.64 ± 0.02 bAB	0.59 ± 0.02 aB	0.65 ± 0.04 bA
	15	0.61 ± 0.03 bAB	0.69 ± 0.03 aA	0.61 ± 0.04 bA
°Brix	0	14.3 ± 0.34 aA	14.3 ± 0.34 aA	12.8 ± 0.35 bAB
	5	14.1 ± 0.10 aA	14.8 ± 0.99 aA	11.9 ± 0.00 bA
	10	14.4 ± 0.10 bA	14.0 ± 0.13 aA	13.9 ± 0.17 aB
	15	15.3 ± 0.19 cB	14.1 ± 0.77 aA	12.1 ± 0.14 bA
ascorbic acid (mg/100 mg)	0	44.86 ± 0.52 aA	44.86 ± 0.52 aA	45.09 ± 0.53 aA
	5	10.52 ± 0.20 cB	12.05 ± 0.21 aB	12.70 ± 0.18 bB
	10	10.33 ± 0.02 bBC	12.57 ± 0.47 aB	12.34 ± 0.17 aB
	15	9.81 ± 0.20 bC	8.65 ± 0.44 aC	10.84 ± 0.85 bC

a Different capital letters show a significant difference (*p* < 0.05) concerning the treatment type. Lowercase letters indicate a significant difference (*p* < 0.05) with respect to the storage time.

b Control fruit was packaged with air and uncoated.

TSS increased by 1 °Brix in control guava shells after 15 days of storage due to the maturation process (respiration). Fruits packaged in MA maintained consistent TSS values, while guavas under EC + MA showed reduced values (approximately 1.5 °Brix, *p* < 0.05) due to the dilution effect of EC. Both MA packaging and EC + MA minimized the ongoing conversion of starch and acid metabolism into sugars during storage. Higher TSS increases (2–3 °Brix) were reported in fresh-cut guava coated with flaxseed protein + guar gum + Tween 40 and uncoated fruit.^16^ Similar behavior was obtained in fresh-cut guava slices coated with potato starch + chitosan after 18 days and control after 6 days at 4 °C.^18^ On fresh-cut pineapple EC + MA packaging, EC and MA packaging slows the TSS increase in a treatment-dependent manner (1.2 to 2 °Brix) during the storage compared with control fruit (2.7 °Brix) at 4 °C.^22^ In addition, in this study, the maturity index (MI) obtained (data not shown) for fresh-cut guavas EC + MA packaging maintained steady values (21.1–19.9), contrasting with control fruit (21.5 to 25.1) during the storage. Thus, EC + MA packaging prevented the loss of quality of fresh-cut guava shells due to the slower fruit gas exchange.

AA content decreased between 72 and 77% after 5 days in guava shells and up to 76–81% at the end of refrigerated storage (15 days). Although significant AA losses were observed in the storage of fresh-cut guava, the EC maintained ∼5% more AA than the control fruit. The AA losses were favored by the cuttings made during the fruit preparation process before being packaged. Peeling and cutting operations deteriorate fresh-cut products due to tissue breakage, increasing respiration and transpiration;^36^ also, O_2_ inside the packaging (10–15%) promotes AA degradation in fresh-cut fruits MA packaged.^37^ Also, protecting effects on AA from EC have been obtained for fresh-cut guava coated with alginate-acemannan (11% more AA than control^2^) and flaxseed protein + guar gum + Tween 40 (12.5% more AA than uncoated^16^). On fresh-cut pineapple cubes, EC, MA packaging, or EC + MA retained between 9 and 21% more AA than the control.^22^

The color of fresh-cut guava shells (luminosity (Lh) and bh parameter (yellowness)) is presented in Figure 2a,b. Both parameters are significantly affected (*p* < 0.05) during the storage. As other researchers mentioned, the EC initially improved the guavas luminosity.^14^ After 5 days of storage, control, and MA packaging, guavas decolored (Lh increase) while Lh of EC + MA increased slightly; decolored could be associated with fruit’s water losses in control and MA packaging while guava shells with EC retained water due to low WVP and WS. Free water content increases because starch hydrolysis produces sugar plus water during maturation.^15,38^ As previously mentioned, EC prevents ripening and retains moisture in guava shells, which leads to color preservation. After 10 days, Lh values dropped and remained steady for up to 15 days. The yellowness (bh) was significantly affected (*p* < 0.05) during refrigerated storage (Figure 2b). Slight yellowness increases were observed after 5 days of storage and were attributed to carotenoid rises. This behavior was similar to those observed by Melo et al.^17^ who measured the β-carotene content in fresh-cut guavas during cold storage (3 °C) and recorded an increase of 1–1.7 μg/g after 6 days of storage. The yellowish tone is a natural indicator of maturity during the ripening process, which exposes carotenoids while chlorophylls are degraded.^15,39^ Afterward, darkening was obtained due to the action of polyphenol oxidase, carotenoid degradation, and microbial growth.^15,17,18,39,40^ Other authors also recorded that luminosity declines on fresh-cut guavas coated or uncoated during storage under refrigerated conditions (3–6 °C).^2^ Similar trends in luminosity (decreases) and protecting effects (EC, MA, and EC + MA) compared with control were observed in fresh-cut pineapple cubes during 16 days of refrigerated storage.^22^

**Figure 2:**
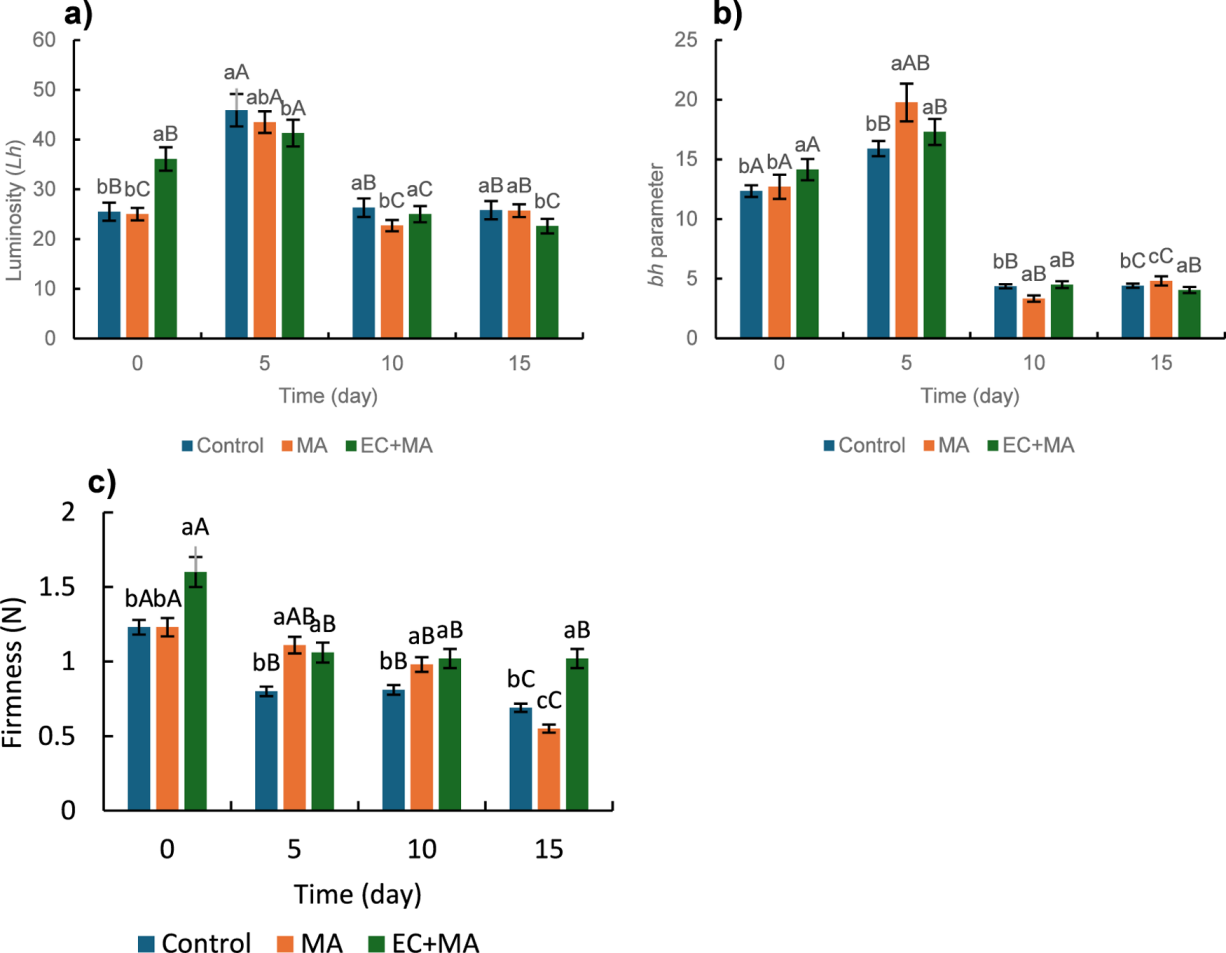
(a) Luminosity (Lh) and (b) yellowness (bh) color parameters and (c) firmness of fresh-cut guava shells in modified atmosphere (MA) packaging and alginate-based coating plus MA packaging (EC + MA) during the refrigerated (5 °C) storage. Control fruit was packaged with air and uncoated. Different capital letters show a significant difference (*p* < 0.05) concerning the treatment type. Lowercase letters indicate a significant difference (*p* < 0.05) with respect to the storage time.

Fruit ripening is usually associated with softening; thus, its firmness is a valuable characteristic of its acceptability. Figure 2c shows the firmness of fresh-cut guava during storage. EC enhanced the guava’s firmness from the beginning to the end of the study. EC provided a continuous layer which protects and improves the texture of guava shells. The SSD evidenced the formation of a thin film covering the whole surface of guava shells. The dipping technique can create thick coatings on the surfaces of fruits.^1,41^ Moreover, AF resistance to puncture (see Section 2.1) protects guava shells integrity whereas enhanced their texture. Calcium ions from EC cross-linking could favor this texture improvement that reached the fruit matrix and interacted with the pectic acids of the fruit’s cell wall.^2^ Control guava shells softened more deeply than those in MA packaging and EC + MA for 10 days; after that, guava shells in MA packaging exhibited lower firmness (*p* < 0.05). The fruit with EC + MA packaging retained 64% firmness, whereas MA packaging and the control only 44 and 56% at the storage end. The progressive softening is related to the pectin enzymatic hydrolysis. Other studies also reported better firmness of coated fresh-cut guava than uncoated fruit at the end of storage (12–18 days) using potato starch + chitosan,^18^ flaxseed protein + guar gum + Tween 40,^16^ and alginate – acemannan EC.^2^ Fresh-cut pineapple cubes with alginate-based coating + MA packaging maintained appropriate firmness than individual treatments (EC or MA) and fruit control during storage,^22^ as occurred in this study.

### 2.5 Pectin-Esterase Activity

Pectin-esterase (PE) is one of the enzymes responsible for the degradation of the cell wall of fruit tissues, which causes fruit softening by reducing intercellular adhesiveness and tissue rigidity.^42^ Table 2 presents the PE enzymatic activity of fresh-cut guava shells during storage. Treatments (MA packaging, EC + MA, or control) had no significant effect (*p* > 0.05) on PE activity during storage. However, marginal increments of PE activity were observed through 15 days of storage, which occurred after 5 days in guavas under MA packaging and the control, while in EC + MA, it was observed after 10 days, which aligns with firmness changes (see Section 2.2). PE activity increases markedly in guava ripening^43^ before decreasing in the overripe stage.^44–46^ However, primary changes occurred between the guava’s mature green and color-turning stages, which remained constant in the ripe stage,^39,47^ which was the stage of fruits in this study. Likewise, García-Betanzos et al.^46^ reported that carnauba wax solid lipid nanoparticles concentration in xanthan gum coating and storage time had no significant effect (*p* > 0.05) on PE activity during the storage of coated guavas. PE activity data from this study and those reported by García-Betanzos et al.^46^ indicated the little effect of coatings on the activity of PE during 15 days of refrigerated storage.

**Table 2:** Pectin-Esterase (PE) Enzymatic Activity in Fresh-Cut Guava Shells in Modified Atmosphere (MA) Packaging and Alginate-Based Coated Plus MA Packaging (EC + MA) Stored at 5 °C^a^ ^,^ ^b^

	PE enzymatic activity (μmeq/g min) × 10^–3^		
time (day)	control	MA	EC + MA
0	0.835 ± 0.005 aA	0.835 ± 0.008 aA	0.929 ± 0.006 aA
5	1.10 ± 0.010 aB	1.160 ± 0.016 aA	0.980 ± 0.011 aAB
10	1.17 ± 0.011 aAB	1.100 ± 0.012 aB	1.31 ± 0.009 aB
15	1.15 ± 0.009 aAB	1.09 ± 0.058 aAB	1.05 ± 0.012 aB

a Different capital letters show a significant difference (*p* < 0.05) concerning the treatment type. Lowercase letters indicate a significant difference (*p* < 0.05) with respect to the storage time.

b Control fruit was packaged with air and uncoated.

### 2.6 Microbial Quality

Figure 3 shows the microbial counts for total mesophilic aerobic bacteria (TMAB, a), yeast and molds (b), and mesophilic anaerobic bacteria (MAB, c) of fresh-cut guava shells packaged during storage. Due to microbial growth, storage time significantly affects microbial counts (*p* < 0.05). TMAB growth was slowed by EC + MA packaging compared with control and MA packaging for 10 days; after 15 days, control exhibited the highest counts (Figure 3a). Yeast and molds gradually grew in EC + MA packaging guava shells for 10 days; afterward, control and EC + MA reached similar counts (Figure 3b). EC may provide carbohydrates as a yeast and mold growth substrate after 5 and 10 days. MAB increased by 3 log_10_ (CFU/g) after 15 days for the three evaluated conditions of fresh-cut guava shells (Figure 3c); a greater increase was observed in MA packaging after 5 days. Subsequently, MAB counts were similar for the three treatments. After 15 days, TMAB in fresh-cut guava shells packaged in MA and EC + MA were below the limit for aerobic plate count (10^6^ CFU/g);^48,49^ therefore, these fruits are safe for consumption after 10 days of storage. Figure 4 displays the fresh-cut guava shells during the storage. In previous reports, higher microbial counts were recorded for fresh-cut guava slices coated with potato starch + chitosan (3 log_10_ CFU/g) for total bacteria counts at day 0, reaching 5.4 log_10_ after 6 days in uncoated fruit and 4.5 log_10_ after 18 days in coated guava slices.^18^ Initial total microbial counts for fresh-cup pineapple cubes ranged from 1.1 to 1.3 log_10_ (CFU/g), reaching 6 log_10_ after 8 days in the control fruit and between 5.5 and 6.4 log_10_ in alginate-based coated fruits, MA packaging, and EC + MA after 14 days of storage.^22^ In other studies, yeast and molds in guava slices, initial counts were 2 log_10_ CFU/g, which grew up to 3.7 and 3.6 log_10_ for the control (after 6 days) and coated (potato starch + chitosan) fruit (18 days), respectively^18^ which were similar or lower counts than those for fresh-cut guava shells of this study. Similar yeast and mold counts were reported by Melo et al.^17^ in fresh-cut guava slices at the beginning (63 CFU/g) and after 12 days of storage 6 × 10^5^ in control and 4 × 10^8^ CFU/g in alginate-based coated guavas. Regarding the shelf life, 1 week is suggested for fresh-cut fruits by 2018–2020 National Advisory Committee on Microbiological Criteria for Foods.^50^ Considering fresh-cut guava shells’ quality and microbial counts, they could be consumed after 10 days of storage when stored under MA packaging or EC + MA.

**Figure 3:**
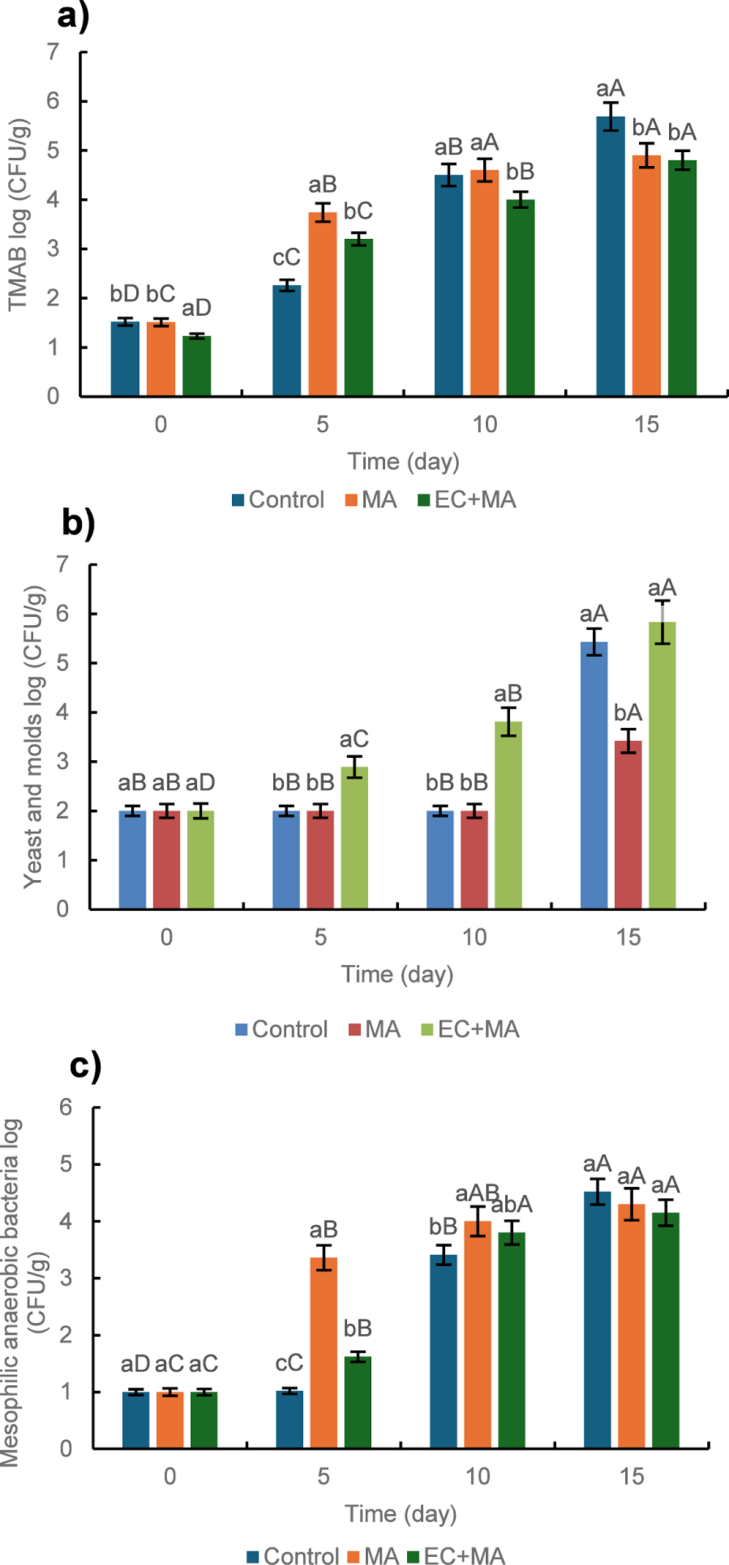
Microbial counts in fresh-cut guava shells in modified atmosphere (MA) packaging and alginate-based coating plus MA packaging (EC + MA) during the refrigerated (5 °C) storage. Control fruit was packaged with air and uncoated. (a) Total mesophilic aerobic bacteria (TMAB), (b) Yeast and molds, and (c) Mesophilic anaerobic bacteria. Different capital letters show a significant difference (*p* < 0.05) concerning the treatment type. Lowercase letters indicate a significant difference (*p* < 0.05) with respect to the storage time.

**Figure 4:**
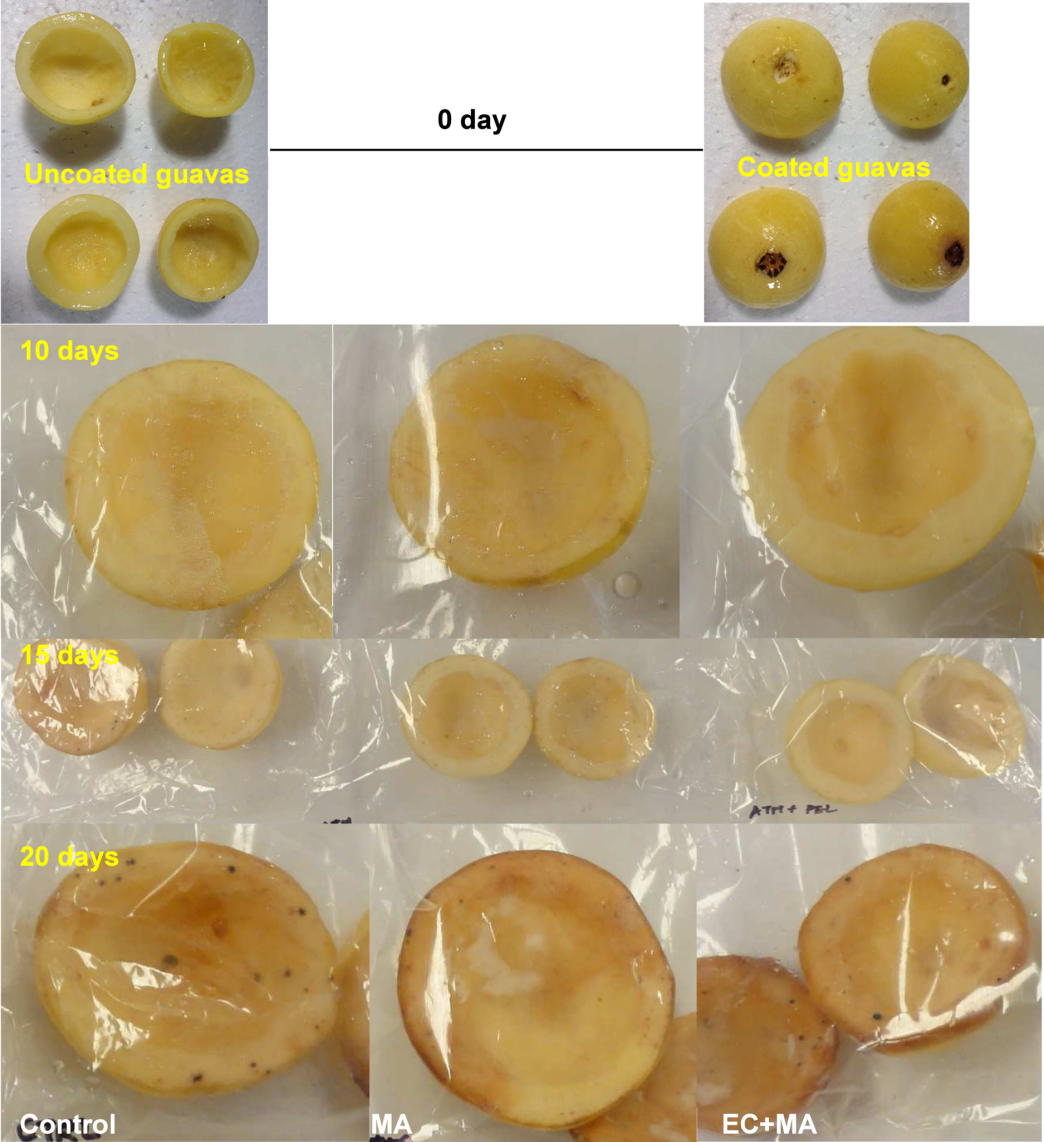
Fresh-cut guava shells during refrigerated storage in modified atmosphere (MA) packaging and alginate-based coating plus MA packaging (EC + MA). Control fruit was packaged with air and uncoated.

Alginate offers a sustainable alternative to conventional synthetic polymers because it is naturally derived, biodegradable, renewable (from brown seaweed biomass), and environmentally friendly. It can also enhance the stability of fresh-cut products fruits. Alginate-EC are useful as a postharvest treatment to maintain fruit quality (preventing microbial growth, lessening respiration rate, and water loss through modulation of fruit respiration and transpiration due to water vapor, O_2_, and CO_2_ barrier), as shown in this study. Thus, alginate-EC has the potential to reduce fruit waste. Moreover, alginate-based coatings align with circular bioeconomy strategies by valorizing marine biomass and minimizing waste. In contrast, the MA packages used in this study are derived from petroleum, which negatively impacts the environment by generating nondegradable waste. However, polymers derived from petroleum are still necessary for packaging since current biopolymers do not satisfy all packaging functions.^51^ Alginate-EC cannot be the end package of fresh-cut guava shells because it is not capable of protecting them from environmental contaminants. According to the results presented in this study, the combination EC + MA packaging improved the guava shells, which is a partially sustainable and environmentally friendly alternative focused primarily on guava shells preservation.

## 3 Conclusions

AF exhibited adequate physicochemical, mechanical, and barrier properties to use as EC. The SSD showed that a continuous film was formed on guava shells. The plastic bags used failed to maintain the gas mixture necessary for modifying the atmosphere during refrigerated storage of fresh-cut guava shells. Despite this, EC + MA packaging slowed the maturation of fresh-cut guava and preserved the AA content better at the end of storage than MA packaging and control fruits. After 10 days of storage, the fruits luminosity was similar to the initial values, whereas yellowness dramatically declined. EC + MA packaging maintained the fresh-cut guava’s firmness for up to 15 days. The PE activity was not affected by MA packaging or EC + MA during storage. Yeast and mold counts reached higher values after 15 days of storage in the control and EC + MA fruits, whereas TMAB were under 10^6^ CFU/g in fresh-cut guava shells packaged in MA or EC + MA. Alginate-based EC maintained physicochemical properties (TA, TSS, AA, luminosity, and firmness) and reduced microbial growth in fresh-cut guava shells during refrigerated storage. Alginate-EC maintains the stability of the fresh-cut guava shells at refrigerated conditions and under MA packaging after 10 days. Therefore, EC + MA packaging improved the postharvest stability of guava, potentially reducing its waste. Further research should consider testing CO_2_ and O_2_ higher barrier plastic bags to evaluate the MA gas mixture for preserving fresh-cut guava and sensory evaluation during storage. Additionally, assessing active-EC could enhance the quality of guava shells, allowing for more extended storage.

## 4 Materials and Methods

### 4.1 Materials

*P. guajava* L. var. criolla was acquired at the central supply center in Puebla, Puebla, Mexico. Fruits were harvested in Michoacan, Mexico (19°24′00.0″N 100°31′48.0″W). Fruits with a typical yellow color, uniform size, and without physical damage were selected. They were sanitized in a sodium hypochlorite solution at 100 mg/L for 2 min. Food-grade sodium alginate (Haugesund, Rogaland, Norway), glycerol (Merck Sharp & Dohme Corp., Whitehouse Station, NJ, USA) as a plasticizer, and calcium chloride (RBM, Puebla, Mexico) to form an insoluble biopolymer were used to form the edible coatings.

### 4.2 Alginate Films Formation and Characterization

Alginate dispersion was prepared with 5 g/L (w/v) alginate in distilled water. The mixture was stirred at 70 °C to achieve complete alginate dispersion. Then, glycerol (6 g/L) was added and stirred for 10 min. Afterward, the mixture was homogenized for 5 min at 24500 rpm and room temperature (∼23 ± 2 °C) using Silverston L4R homogenizer (East Longmeadow, MA) and degassed under vacuum. Twelve mL of film-forming solution was poured into glass Petri dishes (100 × 15) and dried at 60 °C for 6 h. Then, the dried films were peeled off and added with 15 mL of 2% (w/w) CaCl_2_, remaining immersed for 10 min to cross-link the alginate; excess of CaCl_2_ solution was removed, and cross-linked films were dried at 60 °C for 1 h. The films were maintained at an RH of 54% and room temperature (∼23 ± 2 °C) until analysis.

The thickness of five films was measured using a digital micrometer (Mitutotyo Corp., Kawasaki, Japan). The film’s color was determined using a ColorGard System/05 colorimeter (BYK-Gardner Inc., Columbia, MD), which measured the Lh, ah, and bh parameters of the Hunter scale. The hue angle (h) and chroma (C) were then calculated using eqs 1 and 2. Five samples of films were assessed.

1 $$h=arctg\frac{bh}{ah}$$

2 $$C=\sqrt{{ah}^{2}+{bh}^{2}}$$

The mechanical properties, puncture resistance, and elongation percentage were determined following the method ASTM F 1306-90 ^52^ and the methodology described by Soradech et al.,^53^ respectively. The films were fixed on the puncture frame (diameter of 16 mm) and a stainless-steel spherical probe (diameter, 14 mm) coupled to a TA.XT2 texture analyzer (Stable Micro Systems, Godalming, UK) was placed above the film and driven through at a rate of 1 mm/s. The maximum load and the maximum displacement of films were measured. Puncture resistance was determined as the peak force (N) to break the film.^52^ The elongation (%) was calculated using eq 3

3 $$E\left(\%\right)=\left[\frac{\left(\sqrt{r^{2}+d^{2}}\right)-r}{r}\right]\times 100$$

where *r* is the film radius exposed in the cylindrical hole of the film holder, and *d* represents the displacement of the probe from the point of contact to the point of puncture.^53^ Five replicates were analyzed.

The WVP was estimated gravimetrically using a modified version of the standard method ASTM E96–95.^54^ The films were tightened with Parafilm to glass cups (diameter of 25 mm) containing 10 mL of distilled water (headspace of 1 cm). The cups were placed within a desiccator containing saturated MgCl_2_·6H_2_O solution (33% RH) at 25 °C. The water transferred through the film was obtained from the cup’s weight loss over 6 h at 60 min intervals. The water vapor transmission rate (WVTR, g/h m^2^) was calculated with the slope (linear regression) of weight loss versus time divided by the cup’s area (m^2^). Then, eq 4 was used to calculate the WVP as follows

4 $$WVP=\frac{L\times WVTR}{\left(P_{i}-P_{a}\right)}$$

where *L* is the average film thickness (m); *P*_i_ and *P*_a_ are the partial pressures of water vapor in the air and the air saturated to 33% RH at 25 °C.

The water content of films (2 × 2 cm^2^) was analyzed and calculated by weight loss of the films after 24 h at 105 °C. The measurements were performed in triplicate. For water solubility, the percentage of dry films was recorded and used to calculate the percentage of dry films that solubilized after 24 h in distilled water under agitation.^55^

### 4.3 Fruit Coating and Modified Atmosphere Packaging

Sanitized fruits were cut in halves, and seeds were removed to obtain guava shells. Fruits were divided into three batches, as follows: (a) fruit without EC and packaged in atmospheric air (control), (b) fruit packaged in MA, and (c) fruit alginate-coated and packaged in MA. For MA, plastic bags (Polysweat, Grupo Altex, Cd. de Mexico, Mexico) with an oxygen permeability of 4333 cm^2^ /m^2^ day at 23 °C/0% RH, 35 μm, surface area 0.185 m^2^, and a headspace volume of 0.25 L were used. 50 g of fruit were put in each bag and stored at 5 °C and 90 ± 4% RH (20 bags per treatment).

Sodium alginate was dispersed and homogenized for coating fruits as described in Section 4.2. Afterward, guava shells were immersed for 2 min in the film-forming solution, removed for 1 min to eliminate excess liquid, placed into 2% w/w CaCl_2_ solution for 2 min, washed twice in sterile water for 2 min, and set on paper tissue for water removal. A MA equipment (Multivac mod. C100, Sepp Haggenmüller GmbH & Co. KG, Germany) was used to inject the gas mixture of MA consisting of 5.2 ± 0.1% O_2_, 3.9 ± 0.1% CO_2_, and nitrogen (INFRA, Edo. de Mexico, Mexico) into each bag and sealing.

As previously described, the packaged guava shells were analyzed based on their gas composition, physicochemical quality (titratable acidity, soluble solids, ascorbic acid, color, and firmness), enzymatic activity (pectin-esterase), and microbiological quality (yeast and molds, aerobic and anaerobic mesophilic bacteria). The analyses were carried out every 5 days for 15 days, with four packages randomly selected per treatment, and two repetitions were carried out for each one.

The SSD was measured to characterize the coating on guava shells. The guava shells were weighed before and after the coating application to calculate the SSD using eq 5. Then, the EC thickness was obtained from the SSD.^56^ The average area of 20 pieces of guava shells was calculated (10.11 ± 0.49 cm^2^).

5 $$SSD=\frac{M_{Fa}\times X_{s}}{A_{s}}$$

where *M*_Fa_ is the mass of the coating solution adhered to the guava shell (g), *X*_s_ is the mass fraction of solids, and *A*_s_ is the average sample area (cm^2^).

### 4.4 Gas Composition

The MA gas composition inside the packages was determined using an O_2_/CO_2_ headspace analyzer model 902D (Quantek Instruments, Inc., Grafton, MA). Four packages were chosen each time for each treatment, and two repetitions were carried out for each one.

### 4.5 Physicochemical Properties

The color of guava shells was determined on six pieces using a colorimeter ColorGard System/05 (BYK-Gardner Inc., Columbia, MD) measuring Lh, ah, and bh parameters of the Hunter scale in reflectance mode. The instrument was calibrated with a black and white mosaic (*Lh* = 91.89, *bh* = 0.82, *ah* = −1.05). For texture analysis, cylinders (*d* = 2 cm and thickness 0.7 cm) were removed from guava shells utilizing a core borer. Firmness (N) was measured using a texture analyzer TA.TX2 (Stable Micro Systems, Godalming, United Kingdom) and a stainless steel flat cylindrical tip (*d* = 2 mm) at 1 mm/s. For each treatment, 12 cylinders were used for analysis.

The TA was performed following method 942.15 AOAC.^57^ TA was expressed as a percentage of citric acid. TSS were measured with a digital refractometer (Model PR-101, 0–32 °Brix, Atago CO., LTD, Japan) according to the AOAC^57^ method 932.12. AA was determined as described in method 967.21 of the AOAC.^57^

### 4.6 Pectin-Esterase Activity

Guava shells were homogenized in a food processor. The PE activity was measured following the method reported by Rouse and Atkins,^58^ and Salas-Tovar et al.^59^ The release of one μmole of carboxyl groups/min at established conditions is recognized as a pectin-esterase activity unit (PEU). PE activity was determined at pH 7.5 and 35 °C in 1% pectin suspension with 0.2 M NaCl. For sample analysis, 5 g was used, and pH was adjusted every 2 min for 10 min, recording the NaOH volume at each time. To obtain the PEU, a linear regression of NaOH volume versus time was performed, and the slope obtained was used in the following eq (eq 6).

6 $$PEU=\frac{mN}{g}$$

where: PEU are the units of enzymatic activity (μmeq/g min), *m* corresponds to the slope (mL/min), *N* is the normality of the solution, and *g* is the weight of the sample used.

### 4.7 Microbiological Quality

Microbial counts of TMAB, MAB, and yeast and molds were performed during the storage of guava shells. Ten g of guava shells were aseptically removed, transferred to sterile plastic bags (Whirl-Pak, Nasco, Fort Atkinson, WI, USA), and homogenized for 5 min in a stomacher blender 80 (Lab Blender Sewar Ltd., West Sussex, United Kingdom) with 90 mL of sterile peptone water. Then, adequate 10-fold dilutions were made for the analysis. Yeast and mold counts were determined on acidified potato dextrose agar (Bioxon, BD, Edo. de Mexico, Mexico; 1.4 mL/100 mL agar) with tartaric acid 100 g/L, after incubation for 5 days at 25 °C.^60^ TMAB were cultured on standard methods agar (Bioxon, BD, Edo. de Mexico, Mexico) and incubated for 48 h at 35 °C.^61^ For MAB, trypticase soy agar (Bioxon, BD, Edo. de Mexico, Mexico) was used, and plates were incubated under anaerobic conditions at 35 °C for 48 h.

### 4.8 Statistical Analysis

Statistical analysis was performed by ANOVA and Tukey’s mean comparison tests (*p* < 0.05) using Minitab 17 (Minitab, Inc., State College, PA, USA) to identify significant differences in microbial counts, TA, TSS, AA content, PE activity, gas composition, color, and texture.
